# Novel oral chidamide and valganciclovir regimen for EBV-PTLD post-hematopoietic stem cell transplantation: A case report

**DOI:** 10.1097/MD.0000000000048412

**Published:** 2026-04-24

**Authors:** Yaogong Wu, Zhaodong Zhong, Heng Mei

**Affiliations:** a Institute of Hematology, Union Hospital, Tongji Medical College, Huazhong University of Science and Technology, Wuhan, China.

**Keywords:** chidamide, Epstein–Barr virus, hematopoietic stem cell transplantation, posttransplant lymphoproliferative disorder, valganciclovir

## Abstract

**Rationale:**

Epstein–Barr virus-related posttransplant lymphoproliferative disorder (EBV-PTLD) is a serious and potentially fatal complication following allogeneic hematopoietic stem cell transplantation (allo-HSCT).

**Patient concerns:**

We report a case of a 46-year-old male who developed EBV-PTLD 3 months after haploidentical HSCT for refractory acute myeloid leukemia. The patient presented with a persistent cough and a pulmonary nodule that increased in size from 8 mm × 5 mm to 27 mm × 25 mm over 4 weeks. Increased EBV loads were also detected (peripheral blood EBV sorting: EBV-T DNA 2.09E+03, EBV-B DNA 1.16E+03, EBV-NK DNA 7.89E+02 copies/mL; alveolar lavage fluid: 114 sequences; later rising to 3304).

**Diagnoses:**

Diagnosis was confirmed by biopsy which showed EBV-positive pleomorphic lymphoproliferative disorder with extensive necrosis.

**Interventions:**

Treatment consisted of an oral regimen of chidamide (5 mg once daily) and valganciclovir (450 mg twice daily).

**Outcomes:**

After 8 weeks of therapy, the pulmonary lesion decreased to 19 mm × 16 mm, and peripheral blood EBV-DNA levels declined significantly.

**Lessons:**

The patient achieved clinical remission with manageable side effects, including neutropenia and thrombocytopenia.

## 1. Introduction

Allogeneic hematopoietic stem cell transplantation (allo-HSCT) is an effective and sometimes curative therapeutic option for hematological diseases.^[1]^ However, Epstein–Barr virus-related posttransplant lymphoproliferative disorder (EBV-PTLD) is one of the serious complications after allo-HSCT, with an overall incidence of 0.8% to 11.9%.^[2,3]^ The incidence of PTLD varied among different transplant types, with haploid transplantation with the highest incidence of PTLD ranging from 3.4% to 23.5%,^[3]^ and the incidence of PTLD after human leucocyte antigen-matched donor-related and unrelated donors (HLA-MR/UD) ranging from 0.3% to 2.2% and 2.3% to 2.7%,^[4,5]^ respectively. Risk factors after transplantation include grade III/IV acute graft-versus-host disease (GVHD), moderate to severe chronic GVHD, cytomegalovirus (CMV)-DNAemia, and poor CD8+ T cell reconstitution 30 days after transplantation.^[6]^ A recent study has demonstrated encouraging results in the treatment of relapsed/refractory EBV+ lymphoma using a combination of histone deacetylase inhibitor (HDACi) and valganciclovir.^[7]^ Notably, chidamide, the world’s first orally available subtype-selective HDACi, has been approved for the management of relapsed and refractory peripheral T-cell lymphoma (PTCL).^[8]^ This case demonstrates the potential efficacy of a novel oral combination therapy for EBV-PTLD, offering a promising treatment strategy worthy of further investigation.

## 2. Case report

### 2.1. General information

A 46-year-old man with acute myeloid leukemia (AML) with the FLT3-ITD and NPM1 mutation achieved complete remission (CR) with Roxithromycin (DNR, day 1–3)+ cytarabine (AraC, day 1–7)+ etoposide (VP16, day 3), followed by Azacitidine and AraC for 3 cycles. However, he relapsed 5 months later (June 6, 2023), and reinduction therapy with Mitoxantrone, VP16, and AraC. To treat his refractory AML, the patient underwent haploidentical HSCT (haplo-HSCT) using a graft from his 27-year-old daughter’s peripheral blood. The human leukocyte antigen (HLA) match was complete for HVG and partial for GVH, with both donor and recipient having blood type O+. The conditioning regimen consisted of total marrow irradiation combined with VP-16 and cyclophosphamide (Cy). A peripheral blood graft with 7.92 × 10^8^ total nuclear cells/kg and 7.66 × 10^6^ CD34 cells/kg were infused on day 0 (October 10, 2023). The prophylaxis of GVHD included cyclosporin A, methotrexate, mycophenolate mofetil, CD25 monoclonal antibody, and antilymphocyte globulin. Following transplantation, the patient experienced pulmonary and perianal infections. Targeted antimicrobial treatment was initiated, along with symptomatic care. Subsequently, there was an improvement in hematological parameters, meeting the criteria for discharge. A short tandem repeat assay of a bone marrow sample, via polymerase chain reaction, showed complete chimerism. Minimal residual disease detection: no cells with obvious abnormal immunophenotype.

### 2.2. Inspection

**Figure 1.**
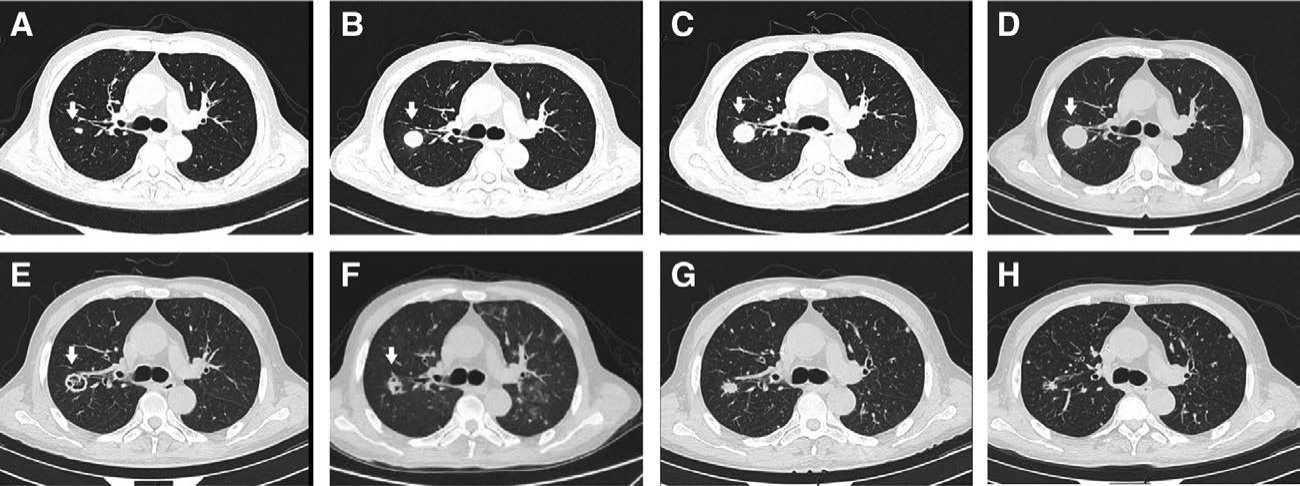
The lung CT scans of the upper lobe of the right lung at different times. (A) December 15, 2023. (B) January 2, 2024. (C) January 11, 2024. (D) January 25, 2024. (E) March 4, 2024. (F) April 3, 2024. (G) June 18, 2024. (H) October 10, 2024. CT = computerized tomography.

On December 15, 2023, the patient was admitted to the hospital with “cough and sputum for 1 week after 3 months of hematopoietic stem cell transplantation for acute leukemia.” Complete blood count: white blood cell (WBC) 1.84 × 10^9^/L, hemoglobin (Hb) of 91 g/L, platelet (PLT) 66 × 10^9^/L. C-reactive protein (CRP) was 33 mg/L, procalcitonin (PCT) < 0.05 ng/mL. The 1,3-β-d-glucan, BDG test (G test) + galactomannan (GM) test was negative. Plasma EBV-DNA was not detected, and intracellular EBV-DNA was 2.03E+03 copies/mL. A computerized tomography (CT) scan of the lungs showed that the posterior segment of the upper lobe of the right lung (Im172) was a solid nodule of about 8 mm × 5 mm (Fig. 1A), which was newer than before; hence, he received treatment with cefoperazone sodium and sulbactam sodium, amphotericin B liposome injection, and posaconazole orally. But the CT scan displayed that the solid nodule of the right lung had increased in size to about 19 mm × 20 mm (Fig. 1B) on January 2, 2024. So the patient underwent fiberoptic bronchoscopy, and next generation sequencing (NGS) of alveolar lavage fluid showed that the number of Epstein–Barr virus (EBV) sequences were high (114). However, the CT scan showed that a solid nodule of about 27 mm × 25 mm (Fig. 1C) on January 11, 2024. To identify what the solid nodule was, with the assistance of an interventional radiologist, a percutaneous needle aspirated biopsy of the right lung posterior upper lobe mass tissue. Pathological results showed EBV-positive pleomorphic lymphoproliferative disorder with extensive necrosis after transplantation (Fig. S1, Supplemental Digital Content, https://links.lww.com/MD/R712). Furthermore, NGS showed EBV with sequence number 3304. Whole-body glucose metabolism PET/CT scintigraphy: heterogeneous nodules in the posterior segment of the upper lobe of the right lung, abnormally elevated metabolic rings, and no obvious signs of malignant tumor lesions were found at the remaining detection sites (Fig. S2, Supplemental Digital Content, https://links.lww.com/MD/R712). Peripheral blood EBV sorting: EBV-T DNA 2.09E+03, EBV-B DNA 1.16E+03, EBV-NK DNA 7.89E+02.

### 2.3. Diagnosis

EBV-PTLD after hematopoietic stem cell transplantation (pulmonary).

### 2.4. Treatment and prognosis

**Figure 2.**
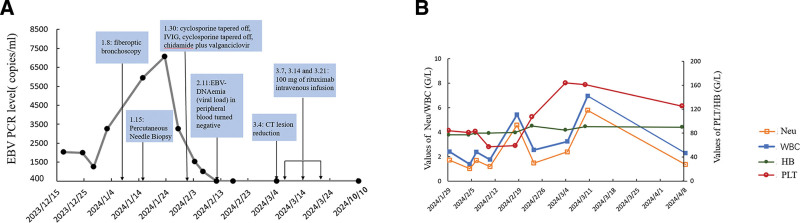
(A) The clinical course of EBV-DNA PCR levels in the present case with markings indicating the timing of chidamide plus valganciclovir, rituximab, and maintenance therapy; (B) peripheral blood cell counts of the patient. EBV = Epstein–Barr virus, HB = hemoglobin, PLT = platelet, WBC = white blood cell.

Given the important results described above, apart from basic treatments such as cyclosporine tapered off, and intravenous immunoglobulin (IVIG), we used a specific therapy which was chidamide (5 mg qd) orally and valganciclovir (450 mg bid) orally (Fig. 2). Encouragingly, the CT scans of the lungs on both January 25, 2024 (Fig. 1D) and March 4 (Fig. 1E) showed the sizes of about 26 mm × 25 mm and 24 mm × 20 mm. Considering EBV sorting results, 100 mg of rituximab intravenous infusion was given on March 7, March 14, and March 21, respectively. So far, the patient’s symptoms are gradually disappearing, with regular treatment and reexamination underway. His CT scan on April 3, 2024 (Fig. 1F) showed a solid nodule of about 19 mm × 16 mm, which subsequently shrank (June 18, 2024, Fig. 1G, 11 mm × 14 mm; April 22, 2025, Fig. 1H, 9 mm × 12 mm).

### 2.5. Ethical approval

The studies involving humans were approved by the ethics committee of Union Hospital, Tongji Medical College, Huazhong University of Science and Technology. The studies were conducted in accordance with the local legislation and institutional requirements. The participants provided their written informed consent to participate in this study.

## 3. Discussion

EBV-PTLDs are a rare and severe entity when occurring after allo-HSCT.^[9]^ So far, the treatment of EBV-PTLD can consist of the reduction of immunosuppression (RI), rituximab, adoptive immunotherapy (EBV-specific cytotoxic lymphocyte or donor lymphocyte infusion), combination chemotherapy, and other approaches. But, these strategies have not been fully effective. Here, we discuss a case in which an EBV-PTLD patient was successfully treated with chidamide and valganciclovir. Moreover, we provide an overview of the associated literature.

EBV-PTLD is a rapidly progressive disease that can give rise to multiple organ failure and death if not treated efficiently. The majority of EBV-PTLD cases are attributable to the outgrowth of donor B cells, with NK and T cells rarely contributing significantly to this disease.^[10]^ For subtypes involving NK or T cells, rituximab cannot be used due to the lack of a cluster of differentiation (CD) 20 target, and treatment is limited. The differentiation toward NK cells could be related to the secreted cytokines such as interleukin-10 and INF-γ.^[11]^ Furthermore, for special types of EBV-PTLD, such as extranodal lesions and central nervous system involvement, the prognosis of patients is poor and treatment methods are relatively lacking. Given that EBV sorting and pathological results (pulmonary, PTLD), the patient in this case received treatment with chidamide and VGCV, apart from basic treatments such as cyclosporine tapered off, Intravenous infusion of human immunoglobulin, and rituximab. Significantly, both EBV-DNAemia (viral load) in peripheral blood (Fig. 2A) and the size of lung lesions are improving (Fig. 1).

Recently, the nanatinostat plus valganciclovir regimen for treating relapsed/refractory EBV+ lymphoma has shown encouraging results.^[7]^ Nanatinostat is a potent, orally administered hydroxamic acid-based class I-selective HDACi highly selective for HDAC 1 to 3.^[12]^ Until now, multiple studies have validated preclinically that EBV lytic cycle activation with HDACi in combination with ganciclovir (GCV) as an effective approach to eliminating EBV-infected tumor cells has a strong mechanistic rationale.^[13-15]^ Haverkos et al reported that Nanatinostat plus valganciclovir was a novel oral regimen for relapsed/refractory EBV+ lymphoma that warrants further investigation in a phase 1b/2 study, in which the overall response rate (ORR) for 43 evaluable patients 40% (n = 17/43), a complete response rate (CRR) of 19% (n = 8/43) and a median time to response of 1.8 months (33–162 days).^[7]^ Similarly, chidamide is also an oral novel isoform-selective HDACi.^[8]^ In addition, a prospective study has revealed the feasibility and safety of chidamide maintenance therapy after allo-HSCT in patients with T-cell acute lymphoblastic leukemia/T-cell lymphoblastic lymphoma.^[16]^ Hence, the patient in this case was treated with chidamide and VGCV. The patient exhibited a good response and achieved remission. Haverkos et al reported that HDACi in combination with VGCV therapy was generally well-tolerated, and the most frequent adverse events (AEs) of any grade were nausea (38%), thrombocytopenia (36%), neutropenia (35%), anemia, and constipation (both 31%).^[7]^ The efficacy of this new combination therapy may be related to the regulation of autophagy in tumor cells.^[17]^ The patient reported treatment-related AEs, which were moderate thrombocytopenia, neutropenia (Fig. 2B), and constipation. Meanwhile, the patient’s immune status did not change significantly (Fig. S3, Supplemental Digital Content, https://links.lww.com/MD/R712).

Important risks and limitations must be emphasized for this case report. The combination poses significant synergistic myelosuppression (e.g., neutropenia, thrombocytopenia), increasing risks of infection and bleeding, alongside potential renal and gastrointestinal toxicities. Long-term safety in immunocompromised patients – such as the impact on immune reconstitution, epigenetic regulation, and secondary malignancy risk – remains unknown. As a single-case report, this study lacks a control group, cannot exclude confounding factors (e.g., concurrent rituximab), and is susceptible to interpretation bias. Future randomized studies comparing this regimen to standard therapies, along with long-term follow-up, are critical to assess its true clinical value and safety profile.

## 4. Conclusion

In this report, we first present the case of an EBV-PTLD patient after allo-HSCT who responded favorably to a novel therapeutic regimen consisting of chidamide and VGCV. In conclusion, the targeted therapy combining chidamide with VGCV holds significant promise as an innovative approach. However, additional clinical cases and in-depth studies are warranted to substantiate our findings and establish the efficacy of this regimen in the management of EBV-PTLD.

## Author contributions

**Conceptualization:** Zhaodong Zhong, Heng Mei.

**Data curation:** Yaogong Wu.

**Formal analysis:** Yaogong Wu, Zhaodong Zhong, Heng Mei.

**Methodology:** Zhaodong Zhong, Heng Mei.

**Writing – original draft:** Yaogong Wu.

**Writing – review & editing:** Yaogong Wu, Zhaodong Zhong, Heng Mei.

## Supplementary Material


