# Supplementary material for: Novel oral chidamide and valganciclovir regimen for EBV-PTLD post-hematopoietic stem cell transplantation: A case report
Source: Medicine (Baltimore). 2026 Apr 24;105(17):e48412. doi: 10.1097/MD.0000000000048412 (PMC13124326; doi:10.1097/MD.0000000000048412)
Supplement: Supplementary file 1 [file medi-105-e48412-s001.pdf]

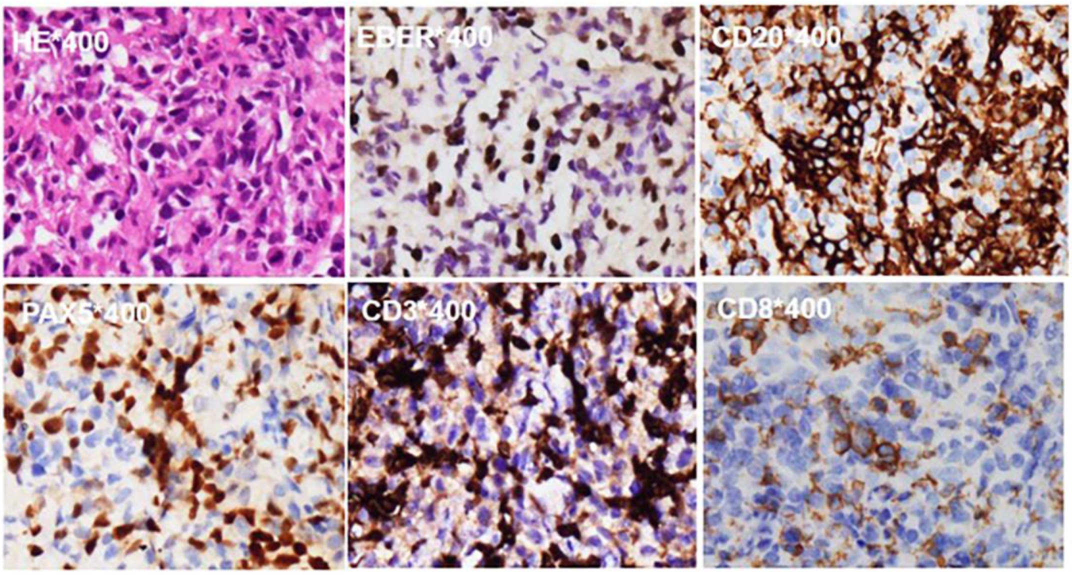

Figure S1. Pathological and immunohistochemical results from right lung upper lobe samples at the time of initial diagnosis. Immunohistochemical staining showed that the larger cells were positive for PAX5, CD20, the smaller were positive for CD3, a few CD8 cells were positive, and EBV was partially positive by in situ hybridization.

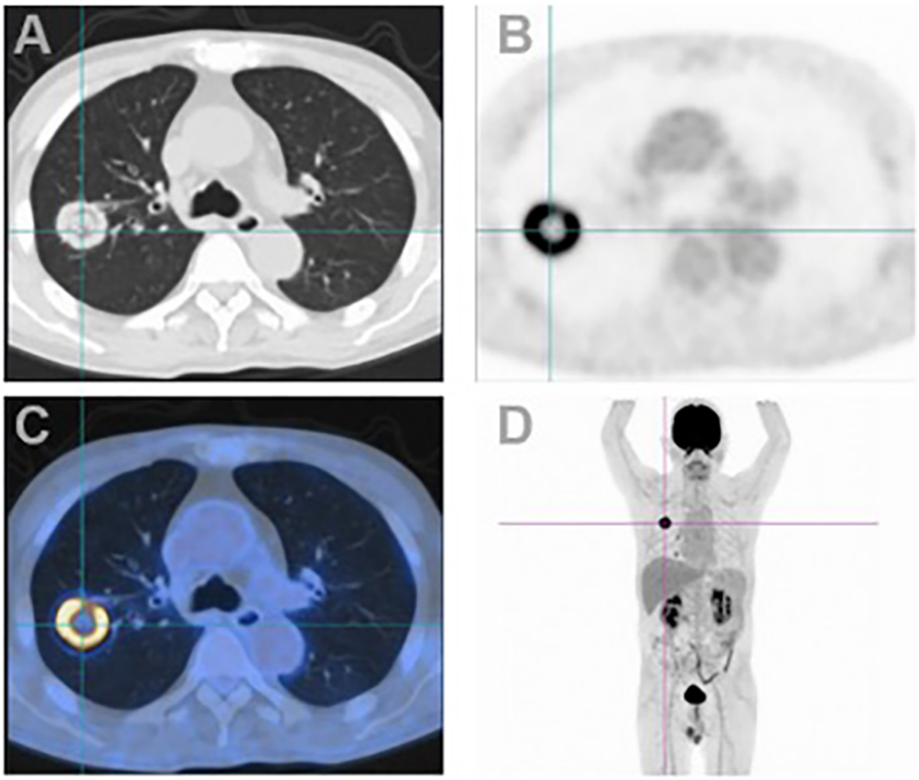

Figure S2. PET-CT revealed that heterogeneous nodules in the posterior segment of the upper lobe of the right lung, abnormally elevated metabolic rings, and no obvious signs of malignant tumor lesions were found at the remaining detection sites.

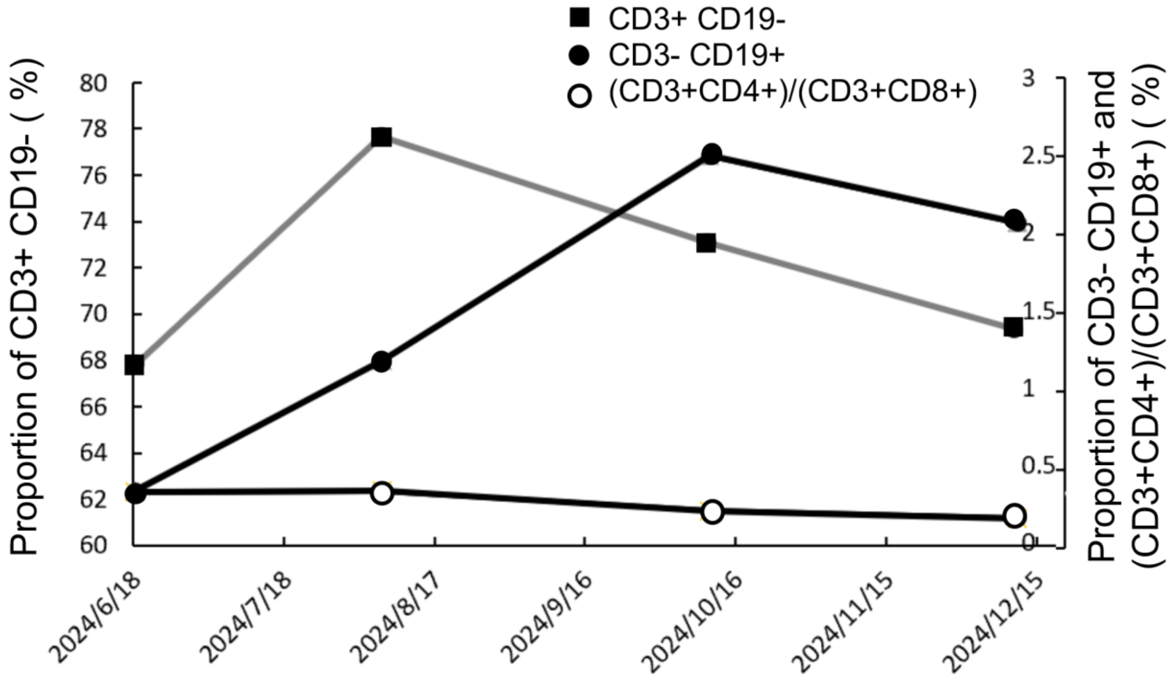

Figure S3. CD3+CD19- cells represent total T lymphocytes, CD3-CD19+ cells represent total B lymphocytes, CD3+CD4+/CD3+CD8+ represents the patient's immune status.
